# A comparative study on the validity and reliability of anterior, medial, and posterior approaches for internal fixation in the repair of fractures of the coronoid process of the ulna

**DOI:** 10.1186/s40001-018-0336-7

**Published:** 2018-09-11

**Authors:** Hong-Wei Chen, Xiao-Feng Teng

**Affiliations:** 1 Department of Orthopedic Surgery, Yiwu Central Hospital, Affiliated Hospital of Wenzhou Medical University, No. 699, Jiangdong Road, Yiwu, 322000 Zhejiang People’s Republic of China; 2 Department of Orthopedics, Ningbo NO.6 Hospital, Ningbo, 315040 People’s Republic of China

**Keywords:** Internal fixation, Coronoid process, Fracture, Posterior approach, Anterior approach, Medial approach

## Abstract

**Background:**

The coracoid process plays an important role in maintaining the stability of the elbow joint. A fracture of the coronoid process is often treated via surgical approaches, including open reduction and internal fixation, which aim to regain a stable, flexible, and loadable joint. In this study, we compared the anterior, medial, and posterior approaches of internal fixation in the repair of fractures of the coronoid process of the ulna.

**Methods:**

In this retrospective study, 147 patients with fractures in the coronoid process of the ulna were recruited and classified into the anterior group (*n* = 73), the medial group (*n* = 32), and the posterior group (*n* = 42) according to the surgical approach used for internal fixation. These patients were assessed with respect to incision, operative time, estimated blood loss, fracture healing, and postoperative complications. The Mayo Elbow Performance Score was used to evaluate any form of disability associated with elbow injuries. Multivariate logistic regression analysis was performed to investigate the factors influencing the efficacy of fractures of the coronoid process of the ulna.

**Results:**

In the medial approach group, the operative time was longer, and perioperative blood loss and postoperative drainage volume were obviously increased compared with the anterior and posterior groups. The anterior group exhibited a better postoperative recovery compared with the medial, and posterior groups. Compared with the anterior group, fracture-healing time in the posterior group was further reduced, whereas elbow joint flexion extension and forearm rotation degree improved. Complications were significantly reduced in the posterior approach group compared with the anterior and medial groups. The factors influencing the efficacy of fractures of the coronoid process of the ulna included the Regan–Morrey classification, perioperative blood loss, and the internal fixation approach.

**Conclusion:**

In summary, the approach used influences fracture healing or the outcome after osteosynthesis. The posterior internal fixation method produced satisfactory functional outcomes in patients with fractures of the coronoid process of the ulna.

## Background

The elbow joint is the second-most common joint in the body that is prone to dislocation in adults. This type of dislocation is classified as simple or complex. A simple dislocation is a dislocation without fracture, whereas complex fracture dislocation is accompanied by a fracture, including radial head fracture, olecranon fracture, or coronoid fracture [1]. In patients with fracture dislocation of the elbow joint, the incidence of radial head fracture was 36%, and 13% of these cases involved a coronoid fracture [2]. The coronoid process is important for the stability of the elbow joint [3]. Given that this condition is often misdiagnosed or ignored due to the complicated pathogenesis, a fracture of the coronoid process of the ulna is a major problem in elbow joint trauma and instability [4]. In previous experiments, Regan–Morrey distinguished three types of coronoid process fractures: type I fracture (the avulsion fracture of the ulna coronal apex), type II fracture (the fracture block does not involve greater than 50% of the entire coronoid process), and type III fracture (the fracture block exceeds 50%) based on the involvement of the coronoid process [5–7].

Various approaches are available in the surgical treatment of fractures of the coronoid process of the ulna, including anterior, posterior, and medial approaches; each of these approaches is associated with advantages and disadvantages [8]. However, some controversy remains regarding the modalities for the treatment of internal fixation of coronoid process fractures in published articles and published surgical procedures [9]. Other experimental data indicate that if the surgical treatment is selected as the treatment of choice for coronoid process fractures, the radial head fracture and lateral collateral ligament complex should be restored prior to the surgery; moreover, whether these fractures need to be fixed to increase external fixation and repair of medial collateral ligament injury should be given special attention [10]. Given that different surgical approaches and fixation techniques are associated with advantages and disadvantages, the present study was conducted to compare anterior, medial, and posterior approaches for internal fixation in the repair of fractures of the coronoid process of the ulna with the aim of providing a reference for a better and safer choice for the treatment of fractures of the coronoid process of the ulna.

## Methods

### Ethics statement

The study was approved and supervised by the Ethics Committee in Yiwu Central Hospital, Affiliated Hospital of Wenzhou Medical University. All subjects recruited for the study signed informed consent forms.

### Study subjects

In total, 147 patients suffering from fractures of the coronoid process of the ulna were selected from the Department of Orthopedics in Yiwu Central Hospital, Affiliated Hospital of Wenzhou Medical University, between January 2011 to May 2017 and were classified into the anterior group (*n* = 73), the medial group (*n* = 32), and the posterior group (*n* = 42) based on the method of internal fixation they received. According to the Regan–Morrey classification, 90 cases were classified as type II coronoid process fracture, and 57 cases were classified as type III coronoid process fracture [11]. The following patients were included: (1) all patients were diagnosed with coronoid process fracture of the ulna using X-ray photography or computed tomography (CT); (2) patients with fresh unilateral fractures of the coronoid process of the ulna who received no treatment; (3) patients who were in good condition and conformed to the indications of surgical treatment; and (4) patients who were cooperative with the terms of the study. The following patients were excluded: (1) patients who underwent an operation on their elbow joint; (2) patients who had an old fracture, open fracture, or comminuted fracture with free articular fragments that could not be corrected through a minimally invasive approach; (3) patients who experienced previous dysfunction of the elbow joint; (4) patients who presented with other complications and injuries and were not able to receive normal fracture treatment; (5) patients with hypertension, diabetes, or other systemic diseases; (6) patients with complicated fractures in other locations in addition to the elbow; and (7) patients who experienced localized necrosis or severe infection.

### Anterior, medial, and posterior surgical approaches for internal fixation

All patients underwent routine examination before the operation, including assessment of anesthetic and operation risks, main biochemical indicators, and positioning of the imaging examination. Following the venous inhalation of compound anesthesia or brachial plexus anesthesia, the C-arm X-ray apparatus was aimed at the elbow joint and adjusted to a suitable angle for traction reduction under the monitoring of the C-arm X-ray apparatus. The entry approach was chosen according to the patients’ fracture condition and the presence of other structural injuries of the elbow joint. The medial approach was suitable for patients with a comparatively large fracture fragment or complete non-comminuted fracture or patients who previously received a simple coronoid fracture resection. The anterior approach was suitable for comminuted or lateral coronoid fracture. The posterior approach was suitable for patients with severe coronoid fracture with complications, including structural injuries on other elbow joints, such as coronoid process fracture complicated with fracture of the capitulum radii and elbow joint dislocation (terrible triad of the elbow) or coronoid process fracture complicated with fracture of the capitulum radii and olecranon fracture. Type II coronoid process fractures were replaced via hand under direct vision and were temporarily fixed with towel forceps or Kirschner needles. A 2-mm-diameter (1–3) lag screw was used to fix and repair the anterior capsule from front to back or back to front. Type III fractures were fixed with Kirschner needles, or the anterior capsule was repaired with non-absorbable sutures. The radial head was resected for patients with comminuted fractures, compression fractures, severe articular cartilage injuries, or combined radial neck fractures for those who were not capable of reposition and fixation. If the elbow joint was still unstable, the lateral collateral ligament was repaired. Three different types of internal fixation methods were performed using the screw plate fixation method. The repair of the anterior bundle of the ulnar collateral ligament was explored during the operation. After the completion of the operation with perspective observation, the incision was sutured layer by layer, and a drain was placed in the wound. The elbow was held in place with a plaster cast for 2 weeks following operation. The patient’s vital signs were monitored, and routine anti-infection therapy was performed after the operation. After 48 h, passive flexion and extension of the elbow joint were performed with the guidance of doctors, and the activities of the active wrists and fingers were assessed. The wound was disassembled 14 days after operation.

### Surgical outcomes

The incision length, operative time, perioperative blood loss, postoperative drainage volume, fracture healing time, and postoperative complications in patients from the anterior group, the medial group, and the posterior group were observed and compared. A lateral side X-ray photograph of the lateral elbow joint was obtained, and fracture healing was evaluated. Lateral elbow joint rotation, flexion, and extension range of motion were measured. Elbow joint pain and the incidence of muscle weakness at 12 months after operation were recorded. Elbow joint function scoring was based on the Mayo Elbow Joint Performance Index [12]: greater than 90 points was excellent, 80–89 points was good, 70–79 points was acceptable, and less than 70 points was poor.

### Statistical analysis

Statistical analysis was conducted using SPSS 21.0 software (IBM Corp. Armonk, NY, USA). The measurement data are presented as the mean ± standard deviation (SD). Comparisons between two groups (homogeneity of variance) were analyzed using the *t* test. The heterogeneity of variance or non-normality was analyzed using the Wilcoxon rank sum test. Counting data were analyzed using the Chi-square test and expressed as a constituent ratio or rate. Logistic regression analysis was used to analyze the factors influencing the surgical efficacy on fractures of the coronoid process of the ulna. *p* was considered as two-tailed probability, and *p* < 0.05 was considered statistically significant.

## Results

### Baseline characteristics of the study subjects

In the beginning of this study, a total of 147 patients with fractures of the coronoid process of the ulnas in the Department of Orthopedics of Yiwu Central Hospital, Affiliated Hospital of Wenzhou Medical University, were divided into the anterior group (*n* = 73), the medial group (*n* = 32), and the posterior group (*n* = 42). The anterior group included 46 males and 27 females with a mean age of 46.4 ± 9.4 years. In this group, 47 cases were classified as Regan–Morrey type II and 26 cases were classified as Regan–Morrey type III. Moreover, 25 males and 7 females were included in the medial group with a mean age of 45.3 ± 8.1 years. This group included 15 Regan–Morrey type II cases and 17 Regan–Morrey type III cases. In addition, 30 males and 12 females were included in the posterior group with a mean age of 44.7 ± 7.2 years, including 28 Regan–Morrey type II cases and 14 Regan–Morrey type III cases. The results revealed no significant differences in mean age, gender, Regan–Morrey classification, injury site, cause of injury, fractures of the coronoid process of the ulna combined with elbow joint dislocation, and the time from injury to surgery among the three groups (all *p* > 0.05) (Table 1).

**Table 1:** Baseline characteristics of study subjects among the anterior, medial, and posterior groups

Subjects	The anterior group (*n* = 73)	The medial group (*n* = 32)	The posterior group (*n* = 42)	p*	*p* ^#^	*p* ^&^
Mean age (years)	46.4 ± 9.4	45.3 ± 8.1	44.7 ± 7.2	0.567	0.313	0.738
Gender (male/female)	46/27	25/7	30/12	0.175	0.417	0.597
Injuries site						
Left elbow joint	28 (38.36%)	10 (31.25%)	15 (35.71%)	0.486	0.843	0.806
Right elbow joint	45 (61.64%)	22 (68.75%)	27 (64.29%)			
Cause of injuries						
Exercise injuries	24 (32.88%)	9 (28.13%)	14 (33.33%)	0.321	0.921	0.581
Falling injury	17 (23.29%)	12 (37.50%)	11 (26.19%)			
Traffic injury	32 (43.84%)	11 (34.38%)	17 (40.48%)			
UCP fractures combined with elbow joint dislocation						
Yes	41 (56.16%)	13 (40.63%)	23 (54.76%)	0.203	> 0.999	0.250
No	32 (43.84%)	19 (59.38%)	19 (45.24%)			
Regan–Morrey classification						
II	47 (64.38%)	15 (46.88%)	28 (66.67%)	0.131	0.842	0.101
III	26 (35.62%)	17 (53.13%)	14 (33.33%)			
Injury to surgery (d)	4.3 ± 1.3	4.2 ± 1.7	4.1 ± 1.4	0.711	0.442	0.747

*UCP fracture* ulnar coronoid process fracture, *n* number

* Comparison of the medial approach and anterior median approach groups; ^#^ comparison of the posterior approach and former median approach groups; ^&^ comparison of the posterior approach and medial approach groups

### Intraoperative outcomes of patients among the anterior group, the posterior group, and the medial group

The intraoperative outcomes of patients were compared among the anterior group, the posterior group, and the medial group. The results demonstrated no significant differences in the incision length among the anterior, medial, and posterior groups (all *p* > 0.05). On the one hand, the operative times, perioperative blood losses, and postoperative drainage volumes of the anterior and posterior groups were significantly reduced compared with the medial group (all *p* < 0.05). On the other hand, slight differences in operative times, perioperative blood losses, and postoperative drainage volumes were observed between the anterior group and the posterior group, but the differences were not statistically significant (all *p* > 0.05). These results revealed that intraoperative outcomes of the anterior and posterior groups might be improved compared with the medial group (Table 2).

**Table 2:** Intraoperative outcomes of patients among the anterior group, the posterior group, and the medial group

Subjects	The anterior group (*n* = 73)	The medial group (*n* = 32)	The posterior group (*n* = 42)	p*	*p* ^#^	*p* ^&^
Incision length (cm)	7.4 ± 1.2	7.8 ± 2.0	7.1 ± 2.1	0.207	0.331	0.151
Operative time (min)	64.0 ± 7.6	73.7 ± 7.3	62.9 ± 7.4	< 0.001	0.452	< 0.001
Perioperative blood loss (mL)	147.1 ± 42.7	171.3 ± 34.6	135.8 ± 44.7	< 0.001	0.182	< 0.001
Postoperative drainage volume (mL)	91.7 ± 7.6	156.3 ± 17.6	94.7 ± 11.4	< 0.001	0.093	< 0.001

*n* number

* Comparison between the medial approach and anterior median approach groups; ^#^ comparison between the posterior approach and former median approach groups; ^&^ comparison between the posterior approach and medial approach groups

### Postoperative recovery of patients among the anterior group, the posterior group, and the medial group

Subsequently, we compared the postoperative recovery based on the anterior approach, medial approach and posterior approach. The anterior, medial, and posterior groups presented significant improvement in incision length and fracture condition. Compared with the medial group, the fracture healing times of the anterior and posterior groups were significantly reduced, the incidence of elbow joint pain was obviously reduced, elbow joint flexion and extension and forearm rotation degree were increased, and the Mayo Elbow Joint Performance Index was distinctly increased (all *p* < 0.05). Moreover, compared with the anterior group, the fracture healing time in the posterior group was further reduced, whereas elbow joint flexion and extension and forearm rotation degree were significantly increased (all *p* < 0.05). However, no significant changes in the incidence of elbow joint pain, muscle weakness, and the Mayo Elbow Joint Performance Index were noted (all *p *> 0.05). These results indicated that the postoperative recovery of the anterior group might be superior compared with the medial and posterior groups (Table 3).

**Table 3:** Postoperative recovery of patients among the anterior group, the posterior group, and the medial group

Subjects	The anterior group (*n* = 73)	The medial group (*n* = 32)	The posterior group (*n* = 42)	p*	*p* ^#^	*p* ^&^
Fracture healing time (week)	12.5 ± 1.3	13.5 ± 1.3	11.0 ± 1.1	< 0.001	< 0.001	< 0.001
Elbow joint pain (yes/no)	19/54	15/17	10/32	0.043	0.828	0.049
Muscle weakness (yes/no)	11/62	9/23	12/30	0.175	> 0.999	> 0.999
Elbow joint flexion/extension (°)	122.2 ± 12.2	111.3 ± 9.3	129.2 ± 9.5	< 0.001	0.002	< 0.001
Forearm rotation (°)	73.4 ± 6.4	68.9 ± 6.1	82.5 ± 8.4	< 0.001	< 0.001	< 0.001
The MEPS	83.5 ± 6.3	80.5 ± 5.5	84.6 ± 7.1	0.022	0.391	0.009

*MEPS* Mayo Elbow Performance Score, *n* number

* Comparison between the medial approach and anterior median approach groups; ^#^ comparison between the posterior approach and former median approach groups; ^&^ comparison between the posterior approach and medial approach groups

### Surgical efficacy of patients among the anterior group, the posterior group, and the medial group

The surgical efficacy among the anterior, medial, and posterior groups was compared. The numbers of patients with excellent, good, acceptable, and bad scores in the anterior group were 45 cases, 22 cases, 3 cases, and 3 cases, respectively. The numbers of patients with excellent, good, acceptable, and bad scores in the medial group were 12 cases, 6 cases, 7 cases, and 7 cases, respectively. The numbers of patients with excellent, good, acceptable, and bad scores in the posterior group were 33 cases, 6 cases, 3 cases, and 0 case, respectively. The excellent rates of the posterior (92.86%) and anterior groups (91.78%) were significantly increased compared with the medial group (56.25%) (all *p* < 0.05). Moreover, no significant differences were noted in the efficacies between the posterior group and the anterior group (*p* > 0.05). These results demonstrated that the efficacies of the anterior and posterior groups were improved compared with the medial group (Table 4).

**Table 4:** Surgical efficacies of patients among the anterior group, the posterior group, and the medial group

Subjects	The anterior group (*n* = 73)	The medial group (*n* = 32)	The posterior group (*n* = 42)	p*	*p* ^#^	*p* ^&^
Excellent	45 (61.64%)	12 (37.50%)	33 (78.57%)	< 0.001	0.836	< 0.001
Good	22 (30.14%)	6 (18.75%)	6 (14.29%)			
Acceptable	3 (4.11%)	7 (21.88%)	3 (7.14%)			
Poor	3 (4.11%)	7 (21.88%)	0 (0%)			

*n* number

* Comparison between the medial approach and anterior median approach groups; ^#^ comparison between the posterior approach and former median approach groups; ^&^ comparison between the posterior approach and medial approach groups

### Incidence of complications of patients among the anterior group, the posterior group, and the medial group

Postoperative complications were recorded after the operation. In the anterior group, 4 cases presented with internal fixation loosening, 8 cases with stiffness or instability of the elbow joint, 6 cases of heterotopic ossification, and 7 cases exhibited other complications. The incidence of complications was 34.25% in this group. In the medial group, 5 patients experienced plate fixation loosening, 3 patients had joint stiffness or instability, 6 patients had heterotopic ossification, and 3 patients had other complications. The complication rate was 53.13% in this group. One patient experienced internal fixation loosening, and 2 patients experienced other complications in the posterior group, and the complication rate was 7.14%. The incidence of complications in the posterior group was significantly reduced compared with the anterior and medial groups (all *p* < 0.05). No significant difference was noted in the incidence of complications between the anterior and medial groups (*p* > 0.05). The above results suggested that the incidence of complications in the posterior group was reduced compared with the anterior and medial groups (Table 5).

**Table 5:** Incidence of complications of patients among the anterior group, the posterior group, and the medial group

Subjects	The anterior group (*n* = 73)	The medial group (*n* = 32)	The posterior group (*n* = 42)	p*	*p* ^#^	*p* ^&^
Plate fixation loosening	4 (5.41%)	5 (15.63%)	1 (2.38%)	0.109	< 0.001	< 0.001
Stiffness/instability of the elbow joint	8 (10.81%)	3 (9.38%)	0 (0%)			
Heterotopic ossification	6 (8.11%)	6 (18.75%)	0 (0%)			
Other complications	7 (9.46%)	3 (9.38%)	2 (4.76%)			
Total	34.25%	53.13%	7.14%			

*n* number

* Comparison between the medial approach and anterior median approach groups; ^#^ comparison between the posterior approach and former median approach groups; ^&^ comparison between the posterior approach and medial approach groups

### Factors influencing the efficacy of fractures of the coronoid process of the ulna

Multivariate logistic regression analysis (LR: backward) was performed using the baseline characteristics of the patients. Intraoperative outcomes and the internal fixation approach were considered as independent variables, and surgical efficacy served as the dependent variable. The results (Table 6) indicate that Regan–Morrey classification, perioperative blood loss, and the internal fixation approach represent factors that influence the efficacy of repair of fractures of the coronoid process of the ulna (all *p* < 0.05).

**Table 6:** Influencing factors for the efficacy of fractures of the coronoid process of the ulna

Variables	B	SE	Wals	Exp (B)	95% CI	*p*
Regan–Morrey classification	2.891	0.644	20.136	18.005	5.094–63.638	< 0.001
Perioperative blood loss (mL)	0.036	0.009	14.568	1.037	1.018–1.056	< 0.001
Approach of internal fixation			8.507			0.014
Anterior group	1.749	0.758	5.326	5.749	1.302–25.396	0.021
Medial group	2.353	0.832	8.000	10.516	2.059–53.702	0.005

*CI* confidence internal

## Discussion

The coronoid process is the anterior portion of the proximal ulnar joint and bone structure that provides frontal humeroulnar stability [13]. Therefore, the central problem that arises from the fracture of the coronoid process involves the injury and elbow joint instability [14]. However, coronoid fractures are typically related to a complex fracture dislocation of the elbow joint, and the incidence of an isolated coronoid fracture is low [9]. It is widely accepted that early surgical treatment is necessary for fractures of the coronoid process of the ulna. Different types of surgical approaches and fixation techniques are available for the treatment of this fracture [15]. Nevertheless, the best and most effective approach has not been identified to date. The key findings from the present study demonstrated that the therapeutic efficacy of fractures of the coronoid process of the ulna treated using the posterior approach of internal fixation is improved compared with the anterior approach and medial approach. This finding could serve as a reference when selecting an approach to surgically treat coronoid fracture surgery.

Our study found that the operative times, perioperative blood losses, and postoperative drainage volumes of the anterior and posterior groups were significantly reduced compared with the medial group, suggesting that the perioperative conditions were better in the anterior and posterior groups compared with the medial group. Moreover, the postoperative recovery of the anterior group was superior to the medial and posterior groups. Surgical treatment of a complex elbow joint injury restores sufficient stability of the elbow joint, providing more rapid improvement in postoperative movement and enhancing elbow joint function [16]. Based on a previous study, successful repair of a bone block is a key factor in the treatment of this challenging injury given that the ulnar coronoid suture significantly improves intraoperative and postoperative stability [8]. Internal fixation is one of the most ideal methods and exhibits good therapeutic efficacy on elbow joint function recovery, thereby making it the main surgical strategy in recent years [17]. In addition, studies have demonstrated that different approaches can be applied to different fracture types. For example, the lateral approach may be the best choice for fractures of the coronoid process of the ulna and radial head fracture, the medial approach is the best choice for ulnar anterior medial surface coronoid process fractures, and the anterior approach is suitable for the Regan–Morrey type III isolated coronoid process fractures [18–20]. Anterior approach fixation is very effective for the treatment of coronal process fracture in the anteromedial ulna. The patients’ postoperative outcomes and Mayo Elbow Joint Performance Index were improved with the use of the anterior approach; however, postoperative elbow cubitus varus and unstable medial rotation delay the recovery time [21].

Furthermore, the incidence of an excellent rating was significantly increased in the posterior and anterior groups compared with the medial group, indicating that the injury was repaired more effectively in the anterior and posterior groups compared with the medial group. In addition, the incidence of complications in the posterior group was significantly reduced compared with the anterior group and the medial group. Although a unified method or surgical internal fixation procedure is not available for the treatment of fractures of the coronoid process of the ulna, previous data have revealed various methods to treat fractures of the coronoid process of the ulna, including arthroscopy-assisted reduction and stable posterior screw internal fixation. Traumatic injuries are traditionally managed with the use of lateral or medial exposure [22]. The combination of the posterior lateral and anterior approaches could improve the activity of the elbow, the Mayo Elbow Performance Score, and visual analog scale results, and the success rate while reducing the postoperative healing time and the complication rate, making this compound approach a clinically effective approach, which was consistent with our findings [23]. However, some defects in the anterior approach were noted, including a very larger diameter of the anterior approach and an increase in bone loss following re-expansion of the operation [18]. Some data indicated that the posterior approach of the elbow is an excellent joint replacement technique [24]. In other experiments, we found that the treatment of tibial plateau condylar fracture with an L-shaped incision via the posterior medial approach offers a full exposure for convenient and effective internal fixation. This method combined with rehabilitation training and restoration of the structure and function of the knee joint provides patients with satisfactory results [25]. Based on the findings of our study, we could infer that the posterior internal fixation approach offers increased safety, reduced postoperative complications and improved efficacy. In addition, multivariate logistic regression analysis showed that the Regan–Morrey classification, perioperative blood loss, and the internal fixation approach are factors that influence the efficacy of fractures of the coronoid process of the ulna.

## Conclusion

In summary, through a comparison of the three internal fixation methods (i.e., anterior, posterior, and medial internal fixation) in the treatment of fractures of the coronoid process of the ulna, we found that the posterior internal fixation approach offers increased safety, reduced postoperative complications, and an improved curative effect, representing the major finding of this study. However, this study is associated with its own limitations. For example, the type of coronoid fracture and relevant elbow injury must be comprehensively considered in the selection of surgical methods and methods of fixation. It is also necessary to further compare the efficacy of multiple and combined approaches on the treatment of fractures of the coronoid process of the ulna to provide a reference for the selection of the internal fixation approach for fractures of the coronoid process of the ulna. Although controversy exists regarding the clinical treatment of fractures of the coronoid process of the ulna, an improvement in the curative effect of fractures of the coronoid process of the ulna was noted using the aforementioned surgical approach.

## Abbreviations

MEPS: Mayo Elbow Performance Score
CT: computed tomography
SD: standard deviation
